# Ubiquitin conjugating enzyme E2 C (UBE2C) may play a dual role involved in the progression of thyroid carcinoma

**DOI:** 10.1038/s41420-022-00935-4

**Published:** 2022-03-24

**Authors:** Cheng Xiang, Hai-chao Yan

**Affiliations:** Department of Thyroid Surgery, The Second Affiliated Hospital of Zhejiang University College of Medicine, Hangzhou, 310009 Zhejiang China

**Keywords:** Thyroid cancer, Prognostic markers

## Abstract

The present study aimed to explore the role of ubiquitin-conjugating enzyme E2 C (UBE2C) in the progress of thyroid carcinoma (THCA). We firstly explored the prognostic impact and expression level of UBE2C in THCA. Then, we performed the UBE2C knockdown and evaluated the effects on the proliferation, cell cycle distribution, apoptosis, migration, and invasion of THCA cells, as well as resistance to sorafenib. Finally, we predicted the possible pathways and explored the correlation between UBE2C with immune infiltrates. The results showed that high expression of UBE2C independently predicted a shorter disease-free survival time of THCA patients. And UBE2C also presented a better prognostic performance on the survival probability of patients. Expression analysis showed that UBE2C was statistically upregulated in THCA tissue compared with normal tissue. After UBE2C knockdown, the proliferation of THCA cells was inhibited and apoptosis was increased. These results indicated that UBE2C acted as an oncogene in THCA. However, the migration and invasion of THCA cells with UBE2C knockdown were enhanced, and the expressions of migration-related proteins were upregulated. In addition, UBE2C knockdown increased the resistance of THCA cells to sorafenib. These results implied the potential of UBE2C as a suppressor gene in THCA. The pathway analysis further predicted that metabolism-related pathways were activated in the UBE2C low expression class, and cell growth and immune-related pathways were focused on the UBE2C high expression class. Finally, we observed a significant positive relationship between UBE2C and several immune infiltrates in THCA. It followed that UBE2C high expression might play a vital role in THCA to some extent. This study revealed that UBE2C participated in the progression of THCA and may play the dual role of both oncogene and tumor suppressor gene. The detailed mechanism needed to be further investigated.

## Introduction

Thyroid carcinoma (THCA) is known as the most prevalent endocrine malignancy and mainly originated from thyroid follicular epithelial cells [1]. The therapies including surgery, radiotherapy, and chemotherapy have been implied in THCA treatment, but they are limited due to drug resistance, postoperative complications, and side effects or unmet efficacy [2]. However, some patients were unresponsive to the therapies such as radioactive iodine [3], which caused disease relapses or even developed to advanced or distant metastatic [4]. Hence, it is necessary to understand the molecular mechanism that drives THCA progression.

UBE2C is a key member of the ubiquitin-conjugating enzyme (E2) family [5] and is located at the nucleus and cytoplasm. It is essential for cell cycle progression, as mutation of the active site (Cys 114 Ser) inhibits the destruction of mitotic cyclins [6]. The expression of UBE2C is cell cycle-regulated [7], as its level is low in G1 [8], accumulates gradually during S and reaches a peak in G2, and then sharply decreases as cells exit from mitosis [9]. Previous evidence has indicated that UBE2C possessed an oncogenic property and the mRNA and/or protein expressions of UBE2C were abnormally upregulated in a wide range of human cancers. High expression of UBE2C caused a poor prognosis of patients in oral squamous cell carcinoma [10], prostate cancer [11], and glioma [12]. In terms of the THCA field, Pallante et al. [13] found that UBE2C was one of the genes most upregulated in THCA cells, and characterized by a highly malignant and aggressive phenotype, whereas it was barely detectable in normal thyroid cells. And this study also indicated its role in thyroid cell proliferation. Eliana et al. [14] found that UBE2C quantitative RT-PCR analysis, rather than immunohistochemistry, increased the detection efficiency of malignancy in thyroid fine-needle aspiration samples. Owing to the importance and promising value of UBE2C in THCA, we further explored the function of UBE2C in the development of THCA based on previous research.

This study firstly explored the prognostic impact of UBE2C in THCA through conducting Kaplan–Meier analysis, Cox regression, receiver operating characteristic (ROC) curve, nomogram, and calibration curve based on bioinformatic analysis. Its expression in THCA was assessed through public data analysis and experimental verification in vivo. To further reveal the functional role of UBE2C in THCA, we detected the influence of UBE2C knockdown on the proliferation, apoptosis, migration, invasion, and drug resistance of THCA cells. The Kyoto Encyclopedia of Genes and Genomes (KEGG) and gene set enrichment analysis (GSEA) analyses were conducted to explore the possible pathways associated with UBE2C. Also, the correlation between UBE2C and immune infiltrates in THCA was detected.

## Results

### The unfavorable prognostic impact of UBE2C in THCA

It has been found that patients with high UBE2C expression suffered a remarkably worse survival rate than those with low UBE2C expression in several human cancers. In this study, we also evaluated the effect of UBE2C on the prognosis of THCA patients. Before the survival analysis, all patients were divided into high and low expression groups according to the median UBE2C expression level. The clinical characteristics of THCA patients were presented in Table 1.

**Table 1:** The clinical characteristics of THCA patients.

Variables	Level	UBE2C low	UBE2C high	*P*
Age	≤55 years old	138	143	0.565
	>55 years old	54	49	
Gender	Male	41	57	0.061
	Female	151	135	
T stage	T1	59	54	0.537
	T2	68	63	
	T3	62	66	
	T4	3	7	
N stage	N0	102	82	0.003
	N1	64	98	
M stage	M0	97	115	0.852
	M1	2	4	
Clinical stage	I	106	121	0.015
	II	29	11	
	III	40	38	
	IV	15	22	
Person neoplasm status	Tumor-free	171	159	0.004
	With tumor	6	22	
DFS status	Disease-free	186	163	<0.001
	Recurred	6	29	
OS status	Living	192	191	0.317
	Deceased	0	1	

*UBE2C* ubiquitin-conjugating enzyme E2 C, *THCA* thyroid carcinoma, *DFS* disease-free survival, *OS* overall survival, *T* primary tumor, *N* regional lymph nodes, *M* distant metastasis.

**Fig. 1:**
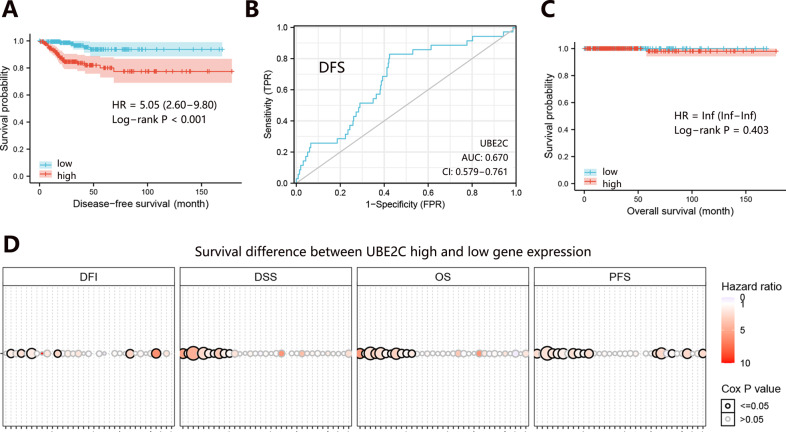
The influence of UBE2C expression on the prognosis of patients. **A** Prognostic impact on DFS of THCA patients. **B** ROC curve for assessing diagnostic value of UBE2C in THCA. **C** Prognostic impact on OS of THCA patients. **D** Prognostic impact of UBE2C among 33 human cancer types. *UBE2C* ubiquitin-conjugating enzyme E2 C, *THCA* thyroid carcinoma, *DFS* disease-free survival, *OS* overall survival, *ROC* receiver operating characteristic.

Then the Kaplan–Meier analysis and Log-rank test were performed to assess the correlation between UBE2C transcript level and survival duration of THCA patients. The results showed that UBE2C high expression was unfavorable to the disease-free survival (DFS) of THCA patients (Fig. 1A). However, the expression of UBE2C did not influence the patient’s overall survival (Fig. 1C). ROC analysis indicated a better prediction performance of UBE2C on DFS of patients (Fig. 1B). Further, we explored the clinical value of UBE2C among 33 human cancer types, finding a consistent unfavorable prognostic impact caused by UBE2C high expression (Fig. 1D). It followed that UBE2C high expression caused a poor clinical outcome of patients in the majority of cancer types including THCA.

To rule out the potential influence of clinical characteristics on the prognosis of THCA patients, Cox proportional hazards regression was further performed. Univariate analysis showed that UBE2C expression, T stage, N stage, M stage, clinical stage, and person neoplasm status were related to DFS of THCA patients (Table 2). Regarding these significant variables, we further constructed a nomogram to evaluate their contribution to predicting survival probability. The result (Fig. S1A) showed that person neoplasm status made the greatest contribution to the survival probability of patients, and the contribution of UBE2C was second only to that of person neoplasm status. The calibration curve of the nomogram was presented in Fig. S1B. ROC curve also indicated the better prognostic performance of UBE2C in THCA (Fig. S1C). Moreover, multivariate analysis indicated that UBE2C mRNA high expression independently predicted the poor DFS of TCHA patients. These results suggested the important prognostic role of UBE2C in THCA.

**Table 2:** Cox regression analysis in terms of DFS in THCA.

Variables	Univariate		Multivariate	
	HR (95% CI)	*P*	HR (95% CI)	*P*
UBE2C mRNA expression	1.813 (1.250–2.631)	0.002	1.238 (0.741–2.069)	0.038
UBE2C methylation level	0.000 (0.000–5.932e + 24)	0.810		
Age	1.107 (0.995–1.039)	0.130		
Gender	0.667 (0.326–1.364)	0.267		
Weight	1.000 (0.998–1.003)	0.900		
T	1.885 (2.255–2.833)	0.002	0.743 (0.390–1.417)	0.368
N	2.567 (1.222–5.394)	0.013	0.991 (0.344–2.855)	0.987
M	6.847 (1.996–23.490)	0.002	0.878 (0.149–5.172)	0.885
Clinical stage	1.679 (1.268–2.223)	<0.001	1.619 (0.964–2.720)	0.069
Person neoplasm status	8.720 (4.298–17.689)	<0.001	7.434 (2.397–23.059)	0.001

*UBE2C* ubiquitin-conjugating enzyme E2 C, *THCA* thyroid carcinoma, *DFS* disease-free survival, *HR* hazard ratio, *CI* confidence interval, *T* primary Tumor, *N* regional lymph nodes, *M* distant metastasis.

### UBE2C was overexpressed in THCA and it correlated with cancer cell growth

**Fig. 2:**
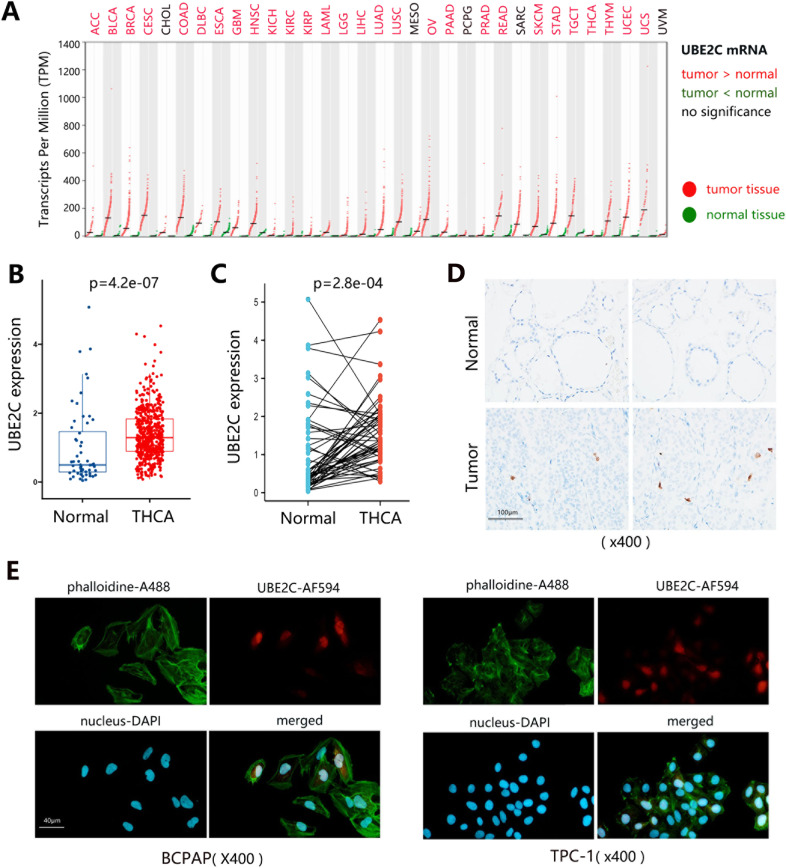
The expression of UBE2C in THCA. **A** The mRNA expression of UBE2C across 33 human cancer types. Each dots represent the expression of samples. The color of the cancer name text indicates the expression difference and statistics. For example, the red color indicated that expression level was statistically higher in tumor tissue than in normal tissue. **B** Differential expression of UBE2C mRNA in normal and THCA samples. **C** Differential expression of UBE2C mRNA in paired samples. **D** Immunohistochemistry verification on protein expression of UBE2C in clinically normal and tumor samples. The blue section indicated the nucleus and the brown or tan section indicated the targeted protein UBE2C. **E** Subcellular location of UBE2C in tumor cells by immunofluorescence. The green, red, and blue fluorescence indicated cytoskeleton, targeted protein UBE2C, and nucleus, respectively. *UBE2C* ubiquitin-conjugating enzyme E2 C, *THCA* thyroid carcinoma.

In order to investigate the role of UBE2C in cancer tumorigenesis, we evaluated the differential expression of UBE2C between normal and cancer tissues. The pan-cancer analysis in Fig. 2A first showed that UBE2C was highly expressed in tumor tissues in almost all cancer types. Combining with the unfavorable prognostic role of UBE2C, it suggested that UBE2C may act as an oncogene in these cancers. Regarding THCA, expression analyses on independent (Fig. 2B) and paired samples (Fig. 2C) indicated the higher expression of UBE2C in the tumor group than in the normal group. We also assessed the UBE2C expression in THCA tissues at the protein level by performing immunohistochemistry and immunofluorescence in vitro to examine the expression pattern (Fig. 2D) and subcellular localization (Fig. 2E).

**Fig. 3:**
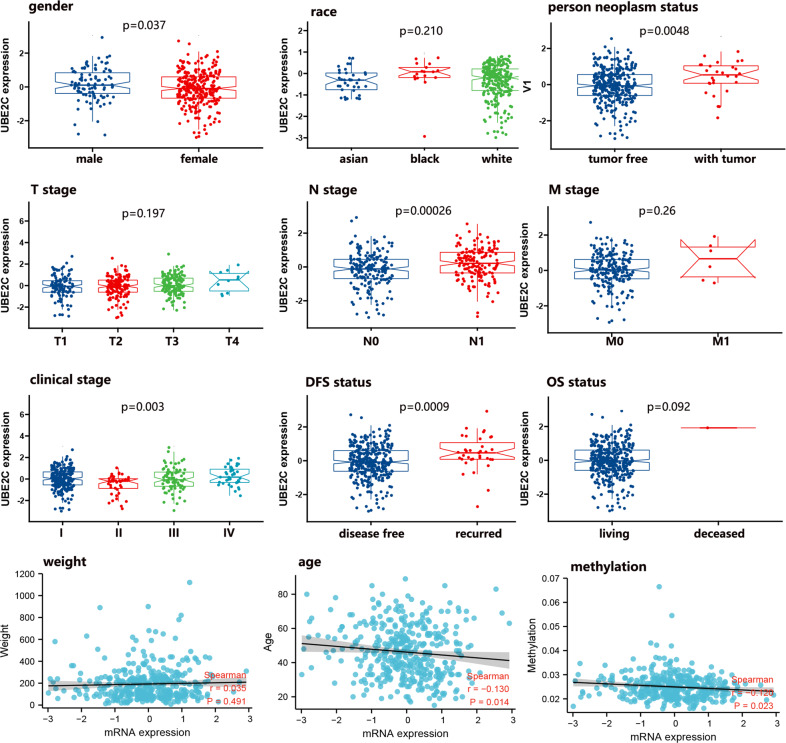
The correlation analysis between UBE2C mRNA expression and clinical characteristics of THCA patients. *UBE2C* ubiquitin-conjugating enzyme E2 C, *THCA* thyroid carcinoma, *DFS* disease-free survival, *OS* overall survival, *T* primary tumor, *N* regional lymph nodes, *M* distant metastasis.

To identify potential associations between UBE2C mRNA expression and clinicopathological parameters in THCA, we compared its abundance among diverse subgroups. We found that the expression of UBE2C was related to the patient’s gender, person neoplasm status, N stage, clinical stage, DFS status, age, and UBE2C methylation level (Fig. 3). But patient’s race, T stage, M stage, OS status, and weight did not influence the UBE2C expression in THCA.

**Fig. 4:**
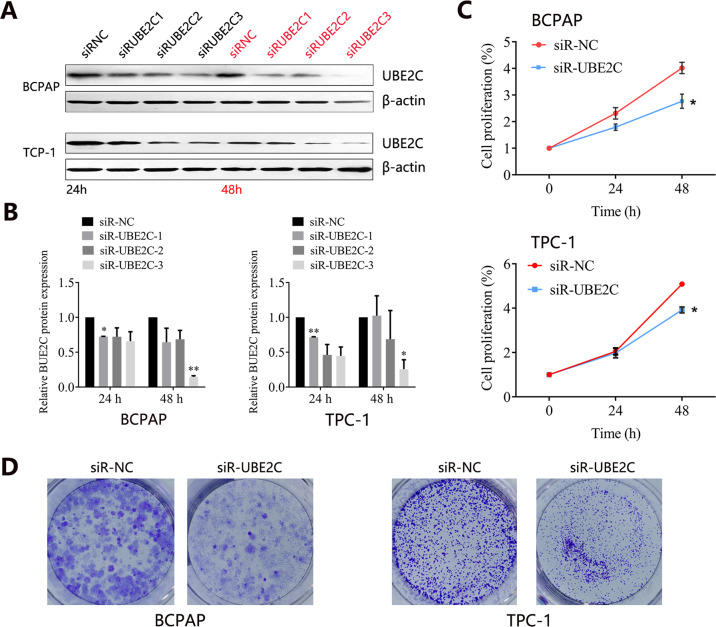
The knockdown treatment of UBE2C in THCA cell lines. **A** Knockdown efficiency determination by western blot. **B** Quantity analysis on UBE2C protein expression with UBE2C knockdown. **C** Effect of UBE2C knockdown with optimal siR-UBE2C-3 on THCA cell growth. **D** Colonies of THCA cells with siR-UBE2C-3 knockdown treatment. *UBE2C* ubiquitin-conjugating enzyme E2 C, *THCA* thyroid carcinoma.

The above results have indicated that UBE2C may play a vital role in the progression of THCA. To investigate the functional role of UBE2C on THCA progression, we transfected the THCA cells with three siRNAs sequences to knock down the UBE2C expression and further confirmed the knockdown efficiency by western blot. The results showed that UBE2C expression was downregulated in THCA cells with three siRNAs transfection for 24 or 48 h (Fig. 4A). Among three siRNA sequences, siR-UBE2C-3 showed the best knockdown efficacy after 48 h transfection both in BCPAP and TPC-1 cells (Fig. 4B). Hence, the siR-UBE2C-3 was selected for further investigation. The MTT assay showed that the cell proliferation ability of THCA cells with UBE2C knockdown was significantly decreased compared with that in siR-NC group (Fig. 4C). Knockdown of UBE2C also decreased the colony formation of BCPAP and TPC-1 cells (Fig. 4D). These results suggested that UBE2C affected the proliferation of THCA cells.

### Effect of UBE2C knockdown on cell cycle distribution, apoptosis, invasion, and migration of THCA cells

**Fig. 5:**
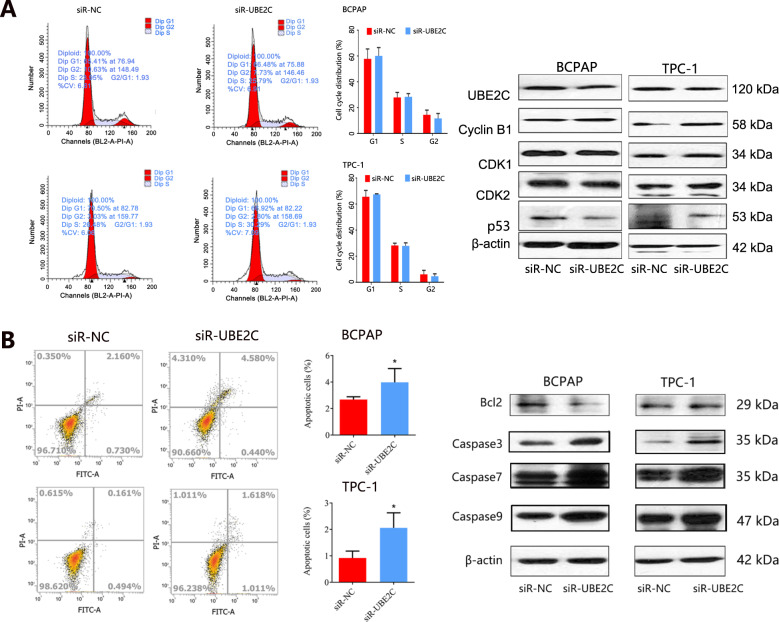
The influence of UBE2C knockdown on cell cycle distribution and apoptosis of THCA cell lines. **A** Cell cycle distribution analysis by flow cytometry and western blot. **B** Cell apoptosis analysis by flow cytometry and western blot. *UBE2C* ubiquitin-conjugating enzyme E2 C, *THCA* thyroid carcinoma.

UBE2C is a key member of the UBE2 family and is located at the nucleus and cytoplasm. The previous study has reported that it was essential for cell cycle progression. We then explored the possible influence of UBE2C on the cell cycle distribution of THCA cells. The results indicated that UBE2C knockdown did not affect the cell cycle distribution of THCA cells (Fig. 5A), but the expression of p53 protein was slightly downregulated in THCA cells. Owing to the inhibitory effect of UBE2C on the proliferation of THCA cells, this study also explored its influence on the apoptosis of cancer cells. Apoptosis assay (Fig. 5B) showed that UBE2C knockdown increased the cell apoptosis both in BCPAP and TPC-1 cell lines. The expression of pro-apoptotic proteins including caspase-3, caspase-7, and caspase-9 were increased both in BCPAP and TPC-1 cells with UBE2C knockdown, whereas the anti-apoptotic protein Bcl-2 was downregulated in THCA cells. It followed that UBE2C knockdown increased the apoptosis of THCA cells.

**Fig. 6:**
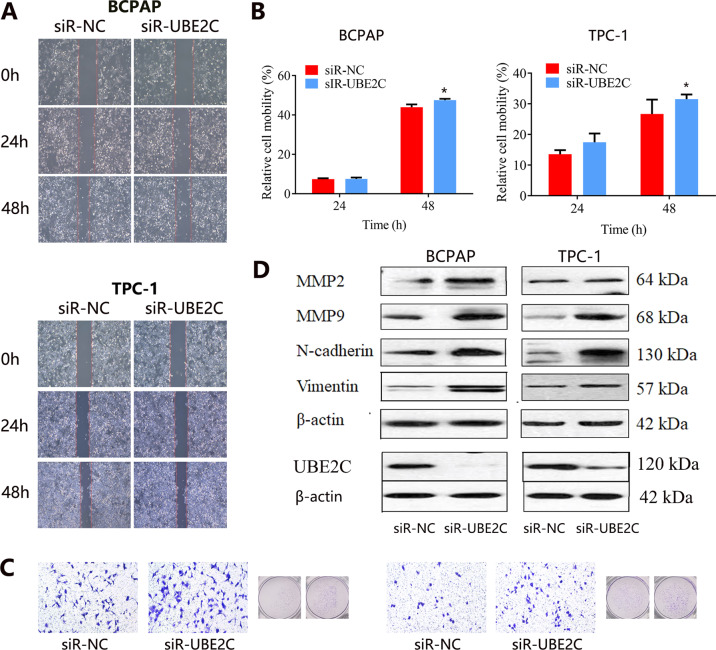
Migration and invasion analyses of THCA cell lines with UBE2C knockdown. **A** Migration ability assessment by wound-healing assay. **B** Quantity analysis on cell mobility. **C** Invasion ability assessment by transwell invasion assay. **D** Western blot analysis on migration-related proteins. *UBE2C* ubiquitin-conjugating enzyme E2 C, *THCA* thyroid carcinoma.

Cellular migration and invasion are critical for cancer metastasis. We then evaluated the effect of UBE2C on the migration and invasion of THCA cells. The wound-healing assay (Fig. 6A) found that the migration ability of THCA cells with UBE2C knockdown for 48 h was increased both in BCPAP and TPC-1 cell lines (Fig. 6B). We also used a transwell assay to detect cell invasion ability. Consistently, UBE2C knockdown also markedly increased the cell invasion ability of THCA cells (Fig. 6C). Western blot analysis indicated that the expression of migration-related proteins of MMP2, MMP9, N-cadherin, and vimentin were all upregulated both in BCPAP and TPC-1 cells with UBE2C knockdown (Fig. 6D). It followed that UBE2C knockdown enhanced the migration and invasion of THCA cells.

### Effect of UBE2C knockdown on the drug sensitivity of THCA cells

**Fig. 7:**
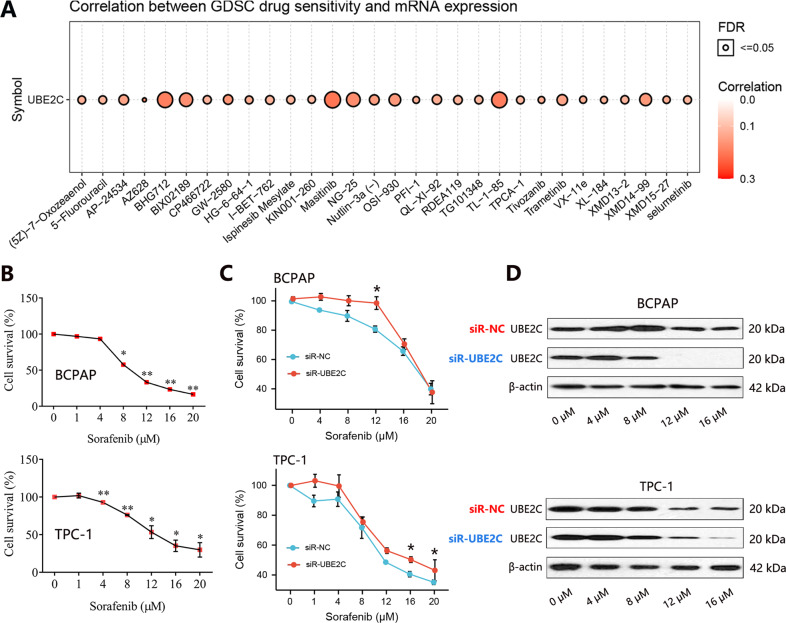
The effect of UBE2C knockdown on the resistance of THCA cells to sorafenib. **A** Correlation between UBE2C expression and CTRP drug sensitivity. **B** MTT was used to detect the cytotoxicity of sorafenib to THCA cells for 24 h. **C** Cytotoxic effect of sorafenib on the THCA cells with UBE2C knockdown. **D** The UBE2C protein expression in THCA cells with UBE2C knockdown was detected by western blot. The THCA cells were treated with different doses of sorafenib.

A recent study demonstrated that UBE2C was involved in chemotherapeutic sensitivity in non-small cell lung cancer cells. And depletion of UBE2C reversed the cisplatin resistance in ovarian cancer. However, whether UBE2C regulates the chemotherapeutic sensitivity of THCA cells remains unknown. We further identified the effect of UBE2C on the chemotherapy resistance of THCA cells. The MTT assay firstly showed that the survival of THCA cells decreased with the increase of sorafenib concentration (Fig. 7B). After the UBE2C knockdown, the survival rate of BCPAP cells in the siR-UBE2C group was higher than that in the siR-NC group when the cells were treated with 12 μM sorafenib (Fig. 7C). Meanwhile, the UBE2C protein expression in the siR-UBE2C group was lower than that in the siR-NC group (Fig. 7D). For TPC-1 cells, the survival rate of cells with 16 μM and 20 μM sorafenib treatment was higher in the siR-UBE2C group than that in the siR-NC group, and UBE2C protein expression in siR-UBE2C group was lower than that in siR-NC group (16 μM). These results suggested that UBE2C expression was positively related to the chemotherapeutic sensitivity of THCA cells. From the GSCA database, the positive correlation between UBE2C expression and drug sensitivity was also confirmed (Fig. 7A). These results indicated the significance of UBE2C on the chemotherapeutic sensitivity in THCA.

### KEGG and GSEA analyses on UBE2C involved in THCA

As the important role of UBE2C on the progression and chemotherapeutic sensitivity in THCA, it was necessary to reveal the potential function associated with UBE2C. We first detected the top 100 co-expressed genes of UBE2C in THCA and further demonstrated the enrichment function of these co-expressed genes by conducting the Gene Ontology (GO) and KEGG analyses. GO analysis in Fig. S2A indicated that co-expressed genes were mainly located at the spindle (CC). The most significant biological process (BP) is referred to as cell division. The primary molecular function (MF) was tubule binding. The KEGG analysis found that co-expressed genes significantly participated in cell cycle and oocyte meiosis pathways (Fig. S2B). It followed that these co-expressed genes were mainly involved in cell growth and death (Fig. S2C). We further performed the GSEA analysis to reveal the detailed regulation of UBE2C on potential pathways in THCA. The analysis in Fig. S3 indicated that UBE2C low expression was mainly related to metabolism-related pathways, such as butanoate metabolism and beta-alanine metabolism. In the UBE2C high expression class, cell growth and immune-related pathways were upregulated.

### Correlation between UBE2C and immune infiltrates in THCA

Above KEGG analysis has indicated the involvement of UBE2C in immune-related pathways in THCA, hence, we explored the correlation between UBE2C and immune infiltrates in THCA. The results showed that most of the immune infiltrate were significantly correlated with UBE2C (Fig. S4A). We further executed the relationships between UBE2C and several immune markers of immune cells, which were widely accepted as corresponding symbols of different immunocytes. The results showed that most of the markers of immune cells were significantly correlated with UBE2C expression (Table 3). In addition, the methylation level of UBE2C was also significantly associated with immune infiltrates in THCA (Fig. S4B). These results showed that UBE2C was closely related to the immune response in THCA.

**Table 3:** Correlation analysis between UBE2C and markers of immune cells in TIMER.

Cell type	Gene marker	Correlation without adjustment		Adjusted by tumor purity	
		cor	*P*	cor	*P*
B cell	CD19	0.394	***	0.406	***
	CD20 (KRT20)	–0.071	0.108	−0.071	0.116
	CD38	0.253	***	0.263	***
CD8+ T cell	CD8A	0.220	***	0.242	***
	CD8B	0.339	***	0.354	***
Macrophage	CD68	0.423	***	0.426	***
	CD11b (ITGAM)	0.447	***	0.45	***
Neutrophil	CD66b (CEACAM8)	0.279	***	0.267	***
	CD15 (FUT4)	0.304	***	0.306	***
Dendritic cell	CD1C (BDCA-1)	0.421	***	0.430	***
	CD141 (THBD)	0.012	0.769	0.030	0.503
	CD11c (ITGAX)	0.468	***	0.478	***

## Discussion

At present, several studies have reported the aberrant expression of UBE2C in human cancers. The frequent overexpression of UBE2C was shown in gastric cancer [15], cervical squamous cell carcinoma [16], and ovarian cancer [17]. More importantly, the UBE2C high expression caused a poor clinical outcome of patients in prostate cancer [11], gastric cancers [18], and lung adenocarcinoma [19]. Through pan-cancer analysis, we found that UBE2C was highly overexpressed in almost all cancer types, and UBE2C high expression caused a poor clinical outcome in the majority of cancers. No favorable prognostic impact of UBE2C was observed. These researches all revealed the oncogenic role of UBE2C involved in cancer progression. To the best of our knowledge, the role of UBE2C in THCA has not yet been thoroughly examined. In this study, we verified that UBE2C mRNA was highly expressed in THCA and high expression caused a shorter DFS time of patients, also suggesting the oncogenic effect of UBE2C in THCA. However, the UBE2C protein staining was weak. The previous study has indicated that even on cell blocks whose corresponding histology was malignant, only a few THCA cells were stained for UBE2C [14]. These results were conceivable because UBE2C expression was limited to cells encompassing the G2/M cell cycle phase, as the research described [20].

We then found that the knockdown of UBE2C inhibited the proliferation and growth of THCA cells, indicating that UBE2C was involved in the progression of THCA. The oncogenic effect of UBE2C in human cancers has been revealed. Several studies showed that depletion of UBE2C blocked cells at the G2 phase [21, 22], and UBE2C was involved in the cell cycle. In this study, we found no disturbing effect of UBE2C knockdown on the cell cycle distribution of THCA cells, but the proliferation of THCA cells was impeded and apoptosis was increased. The KEGG pathway analysis partially supported our results, and KEGG analysis showed that UBE2C co-expressed genes were mainly involved in cell growth and death. The inhibition of proliferation and promotion of apoptosis caused by UBE2C knockdown was also detected in esophageal squamous cell carcinoma [23]. In addition, the knockdown of UBE2C significantly attenuated the migration and invasion in hepatocellular carcinoma cells [24]. UBE2C knockdown also inhibited cell proliferation, migration, invasion, and epithelial-mesenchymal transition (EMT) in endometrial cancer, whereas overexpression exerted the opposite effects [25]. In gastric cancer, interfering with UBE2C expression caused the inhibition of cancer cell proliferation, migration, and invasion as well [26]. Conversely, this study reported an opposite effect, finding that UBE2C knockdown enhanced the migration and invasion ability of THCA cells. The western blot analysis confirmed the upregulation of migration-related proteins including MMP2, MMP9, N-cadherin, and Vimentin in THCA cells with UBE2C knockdown. Our result initially indicated that UBE2C knockdown might promote the EMT process in THCA, which plays a key role in the process of tumor invasion and metastasis. The matrix metalloproteinases (MMPs) are a kind of important proteolytic enzyme, and can effectively degrade ECM and basal membrane [27]. And the increase of MMPs expression was highly correlated with tumor invasion and metastasis. Our results further demonstrated that UBE2C participated in the migration and invasion process of TCHA. It should be mentioned that although interfering with UBE2C expression inhibited tumor proliferation and induced cell apoptosis in THCA, it might pose the risk of tumor migration and invasion, thus inducing the recurrence of the disease.

We further conducted the GSEA analysis to reveal the potential mechanism associated with UBE2C in THCA. We found that metabolism-related pathways were upregulated in the UBE2C low expression group. Hence, we speculated that UBE2C knockdown might upregulate the metabolism pathway, thus promoting the behavior of THCA cells. The previous study identified 31 metabolites referring to amino acid and lipid in PTC samples with distant metastasis, suggesting that serum metabolomics profiling could significantly discriminate PTC patients according to distant metastasis [28]. Budhu et al. [29] identified 28 fatty acids metabolites associated with the progress of liver cancer. The important role of metabolism in the process of tumor migration has been largely revealed. In addition, we also found that UBE2C high expression was significantly related to cell growth and immune-related pathways. In this study, we observed that UBE2C was positively related to several immune infiltrates and their markers. The results indicated the significance of immune infiltrates in THCA.

Increased evidence has proved that the expression of UBE2C could be employed to predict response or resistance to chemotherapy or targeted agents. Knockdown of UBE2C sensitized the cells to pharmacological treatments with irinotecan in colorectal cancer [30] and increased the sensitivity of cancer cells to epirubicin and docetaxel in breast cancer [31]. However, this study also found opposite results. The experiment in vivo demonstrated that the UBE2C knockdown reduced the sensitivity of THCA cells to sorafenib treatment, and increased the survival rate of THCA cells. Through further analysis, we found a positive correlation between UBE2C expression and GDSC drug sensitivity of all chemotherapy drugs. The correlation analysis also conformed to our experimental verification.

Our results found that UBE2C knockdown enhanced the migration and invasion ability of THCA cells. It also reduced the sensitivity of THCA cells to sorafenib treatment. These results differed from other research. There was no reasonable explanation for these different results so far, and we speculated that UBE2C may play different roles in different pathological types of tumor cells, and may exert different effects even within the same tumor. In addition, this study suggested that UBE2C has a very complex and strict regulatory mechanism on the occurrence and development of THCA, and it may play the dual role of both oncogene and tumor suppressor genes in THCA. Therefore, the potential regulatory mechanism of UBE2C in THCA progression still needs to be further investigated.

## Conclusion

This study presented that UBE2C was highly expressed in THCA samples compared with the normal group. The high expression of UBE2C shortened the DFS time of THCA patients and independently predicted the poor prognosis in THCA. Knockdown of UBE2C inhibited the proliferation and increased the apoptosis of THCA cells. Pathway analyses also indicated that UBE2C was involved in cell growth in THCA. It followed that UBE2C was associated with THCA progression and may act as an oncogene in THCA. Unexpectedly, UBE2C knockdown enhanced the migration and invasion ability, as well as reduced the sensitivity of THCA cells to sorafenib. These results implied the potential of UBE2C as a potential suppressor gene in THCA. Pathway analysis presented that the metabolism-related pathways were upregulated in the UBE2C low expression class. This study initially uncovered the unintended consequence, and the potential regulatory mechanism of UBE2C in THCA progression still needs to be further investigated.

## Methods

### The effect of UBE2C on the prognosis of THCA patients

This study firstly evaluated the prognostic value of UBE2C in THCA. We obtained the clinical data of THCA patients and expression profile of UBE2C from the cBioportal database (http://www.cbioportal.org/). The inclusion criteria: (a) samples diagnosed as thyroid cancer; (b) samples with mapped clinical information and gene expression matrix; (c) samples with complete clinical information including survival time, survival status, age, and gender. The exclusion criteria: (a) normal tissue samples; (b) samples without complete clinical information; (c) samples with no expression value; (d) samples with bias in expressional value. And then we assessed the influence of UBE2C expression on DFS and overall survival of THCA patients by performing Kaplan–Meier analysis and log-rank test. The ROC curve was used to assess the prognostic performance of UBE2C on the survival probability of patients. Further, the Cox regression analysis was conducted to rule out the potential influence of clinical characteristics on the prognosis of THCA patients. A nomogram regarding the important variables from Cox regression was constructed to assess the distribution of these variables on the survival probability, followed by calibration curve construction. The ROC analysis on these variables was also performed to evaluate their prognostic performance.

### The expression of UBE2C in THCA

The differential expression of UBE2C mRNA in THCA and normal thyroid tissues was analyzed using the TCGA-THCA data set (https://xenabrowser.net/). The correlation between UBE2C mRNA expression and clinical characteristics of THCA patients was then explored. The clinical characteristics for correlation analysis included patient’s age, gender, race, person neoplasm status, topography (T) stage, lymph node (N) stage, metastasis (M) stage, clinical stage, DFS status, overall survival (OS) status, UBE2C methylation level, and weight. We also detected the protein expression of UBE2C in THCA by immunohistochemistry and its subcellular location by immunofluorescence.

### Cell culture and transfection

The BCPAP and TPC-1 cells were obtained from the laboratory of the pathology of Zhejiang University. The cells were incubated in Dulbecco’s Modified Eagle Medium (DMEM) media, supplemented with 10% fetal bovine serum, 100 U/mL penicillin, and 1× glutamine at 37 °C in a 5% CO_2_ atmosphere. The cells at the logarithmic phase were chosen for further experiment. For small interfering RNA (siRNA) transfection experiments, we transfected the cells with three siRNA sequences (100 nM) targeting UBE2C and negative control siRNA. Table 4 showed three different siRNA sequences for UBE2C knockdown. Total proteins were extracted after siRNA treatment for 48 h, and the protein levels of UBE2C were then detected by western blot to assess the knockdown efficiency.

**Table 4:** Three differential small interfering RNA sequences for UBE2C knockdown.

siRNA	Sense	Antisense
siR-NC	UUCUCCGAACGAGUCACGUTT	ACGUGACUCGUUCGGAGAATT
siR-UBE2C-1	AGUGGUCUGCCCUGUAUGATT	UCAUACAGGGCAGACCACUTT
siR-UBE2C-2	AGGGAUUUCUGCCUUCCCUTT	AGGGAAGGCAGAAAUCCCUTT
siR-UBE2C-3	CCCUUACAAUGCGCCCACAGUTT	ACUGUGGGCGCAUUGUAAGGGTT

### The effect of UBE2C on the cell proliferation of THCA cells

The MTT assay was used to evaluate the proliferation ability of THCA cells. The BCPAP and TPC-1 cells with siRNA transfection were seeded into 96-well plates and cultured with 100 μl cell suspension for 24 h and 48 h. The cells were then incubated with 50 μl MTT solution (1 mg/ml) for 3 h at 37°C. Subsequently, the absorbance at 450 nm was measured using a spectrophotometer.

### The effect of UBE2C on colony formation

The influence of UBE2C knockdown on cell growth was evaluated by colony formation assay. Transfected THCA cells were harvested with proenzyme and then seeded into the six-well plates. The cells were cultured for 10 days and the culture medium was replaced with a fresh medium every 3 days. Finally, the cells were washed with PBS, fixed with 0.8 ml paraformaldehyde for 20 min at 37°C, and stained with 0.8 ml crystal violet for 30 min at 37°C. The number of colonies was observed and counted using an optical microscope.

### The effect of UBE2C on cell cycle distribution of THCA cells

Cell cycle distribution was detected using flow cytometry in THCA cells. The harvest treatment of THCA cells was stopped with a serum medium. Then the cells were washed with pre-cooling PBS and fixed in 70% chilled ethanol at 4 °C overnight. Next, we centrifuged cells at 300 × *g* for 5 min. The cells were subsequently incubated with 500 μl propidium iodide (PI) solution and 2.5 μl RNase for 5 min at room temperature. Finally, the cells were stained with 50 μg/ml PI solution for 30 min at 4°C in the dark. Cell cycle distribution was measured within 24 h by flow cytometry. The expression of cell cycle-related protein (CDK1, CDK2, P53, CyclinB1) was also detected by western blot.

### The effect of UBE2C on apoptosis of THCA cells

The apoptosis of THCA cells was detected by flow cytometry analysis. The cells were harvested and washed with pre-cooling PBS. Then, we resuspended the cells in the binding buffer at a concentration of 5 × 10^5^ cells/ml. Next, the 100 μl cell suspension was incubated with 5 μl Annexin V/FITC for 5 min at room temperature in the dark. Subsequently, 10 μl PI solution (20 μg/ml) and 400 μl PBS was added, and apoptosis of cells was measured by flow cytometry. The expressions of apoptosis-related proteins containing Bcl-2, Caspase-3, Caspase-7, and Caspase-9 were also detected by western blot.

### The effect of UBE2C on migration and invasion of THCA cells

A wound-healing motility assay was performed to evaluate the migration ability of THCA cells. UBE2C knockdown cells (5 × 10^5^ cells/ml) were cultured with a serum-free medium in six-well plates overnight at 37 °C and 5% CO_2_ supply, and a scratch was then made with a 10 μl pipette tip. Next, the cells were continuously incubated for 48 h. We counted the scratch distance and calculated the migration rate.

A Transwell assay was performed to assess the invasion of THCA cells. The 100 μl cell suspension was added to the upper chamber containing 100 μl Matrigel mixed solution. And 500 μl medium with 10% PBS was added into the lower chamber. After incubation for 24 h, the migrated or invasive cells were fixed with methyl alcohol for 20 min and then stained with crystal violet for 40 min. Cell migration and invasion were assessed using an inverted microscope. The expression of the migration-related protein (MMP2, MMP9, vimentin, N-cadherin) was also detected by western blot.

### The effect of UBE2C on resistance to sorafenib of THCA cells

The proper concentration of sorafenib treatment on the THCA cell survival was firstly screened. After transfection with siRNA for 48 h, the effects of sorafenib with different concentrations on the survival of THCA cells were measured by MTT assay. In addition, the UBE2C protein expression in THCA cells with UBE2C knockdown was detected by western blot. Further, we explored the correlation between UBE2C expression and CTRP drug sensitivity in the GSCA database (http://bioinfo.life.hust.edu.cn/GSCA/#/).

### Western blot assay

Western blot was performed as described elsewhere. The antibodies used in this study was presented in Table 5.

**Table 5:** List of antibodies used.

Protein name	Company	Art. no.	Protein name	Company	Art. no.
β-actin	CST	4970	P53	CST	2525
UBE2C	Abcam	ab252940	CyclinB1	Abcam	ab32053
CDK1	Abcam	ab133327	Bcl-2	CST	15071
CDK2	Abcam	ab32147	Caspase-3	Abcam	ab13847
Caspase-7	Proteintech	271555-1-AP	N-cadherin	Proteintech	22018-1-AP
Caspase-9	CST	9502	MMP2	CST	87809 S
MMP9	Abcam	ab76003	Vimentin	CST	5741
Goat anti-rabbit IgG antibody	CST	7074	Goat anti-mouse IgG antibody	CST	7076

### Immunofluorescence

The subcellular localization of UBE2C in THCA cells was detected by immunofluorescence. The cells treated with siRNAs were cultured with 0.5 ml DMEM medium for 24 h. After washing cells with PBS twice, cells were fixed with 4% paraformaldehyde for 20 min at room temperature and 0.1% Triton X-100 was used to perforate the cell membranes for 5 min. Next, the cells were covered with 5% BSA for 90 min at room temperature. After this, cells were incubated with anti-UBE2C (1:50) antibody at 4 °C overnight and then cultured with secondary antibodies for 1 h. Cells were orderly stained with DAPI (1 μg/mL) for 5 min and phalloidine for 20 min. Finally, cells were examined by confocal microscopy using a fluorescein filter.

### Enrichment analysis on UBE2C

We firstly obtained the co-expressed genes of UBE2C in THCA from the cBioportal database. According to the threshold of *P* < 0.05 and absolute correlation coefficient, the top 100 co-expressed genes were selected for further analyses. The potential function of co-expressed genes was assessed by performing GO annotation and KEGG analyses using the R package cluster profile. GO annotation analysis contained the CC, BP, and MF. The KEGG analysis was used to reveal the possible pathways associated with UBE2C co-expressed genes.

Further, we performed the GSEA to reveal the potential mechanism associated with UBE2C involved in THCA. The expression profile of the TCGA-THCA dataset was used for GSEA analysis. According to the median expression of UBE2C, all patients were divided into high and low expression groups. Then we performed the GSEA analysis to predict the pathways referring to the UBE2C low expression or high expression classes. The terms with *P* < 0.05 and false discovery rate < 0.25 were regarded as significant pathways. The Normalized Enrichment Score was used to evaluate the enrichment degree.

### Correlation between UBE2C and immune infiltrates in THCA

Immune infiltration plays a vital role in cancer progression. We explored the relationship between UBE2C and immune infiltrates in THCA by the Pearson method in the GSCA database. Detailed, the correlation between UBE2C and gene makers of immune infiltrates with or without tumor purity adjustment was analyzed in the Timer database (https://cistrome.shinyapps.io/timer/).

### Statistical analysis

The SPSS 23.0 was used for statistical analysis. All the experimental results in this study were obtained from at least three independent repeats. The continuous variables were expressed as mean values ± standard deviation. The two-tailed student’s *t* test or one-way analysis of variance was used to compare the difference between groups of quantitative data. The *χ*^2^ test was used to compare the difference of qualitative data. Kaplan–Meier analysis, Cox regression, nomogram, and ROC curve were used for prognosis-related analysis. Pearson analysis was used to explore the correlation between two variables. *P* value <0.05 was regarded as statistically significant.

## Supplementary information

supplementary figures

## Data Availability

The data that support the findings of this study are available from the corresponding author upon reasonable request.
